# Tracheal rupture after vocal cord polyp resection: A case report

**DOI:** 10.1097/MD.0000000000028106

**Published:** 2021-12-17

**Authors:** Xinqi Hu, Xiaofeng Chen, Xidong Cui, Yitan Cao, Guangbin Sun

**Affiliations:** a Department of Otorhinolaryngology – Head and Neck Surgery, Huashan Hospital of Fudan University, Shanghai, China; b Department of Cardiothoracic Surgery, Huashan Hospital of Fudan University, Shanghai, China.

**Keywords:** case report, laser microsurgery, tracheal rupture, vocal cord polyp resection

## Abstract

**Introduction:**

Transoral laser microsurgery (TLM) is one of the most common operations performed for glottic lesions. Several protection measures are taken to prevent tracheal damage. However, some protection measures and common postoperative complications may still cause delayed tracheal rupture in certain situations. Cases of tracheal rupture after surgery are extremely rare, and there are no previous reports of TLM of the glottis causing tracheal rupture.

**Patient concerns:**

A middle-aged woman who underwent TLM for bilateral vocal cord polyps developed sudden neck pain, followed by cough and subcutaneous emphysema.

**Diagnosis:**

She underwent head, neck, and chest computed tomography (CT), which revealed a 4-cm membranous tracheal tear located 4.5 cm distal to the glottis, pneumomediastinum, and subcutaneous emphysema extending from the base of skull to the chest.

**Interventions:**

The patient underwent an emergency surgical surgical chest exploration and tracheal repair.

**Outcomes:**

One month after the surgery, the patient fully recovered with no tracheal stenosis or respiratory dysfunction.

**Conclusions:**

Conventional protective measures and common postoperative complications of TLM may also cause tracheal rupture.

## 1 Introduction

Tracheal rupture is a serious condition usually caused by neck and/or chest trauma. Cases of tracheal rupture after surgery are extremely rare, and most cases occur only during bronchoscopic or esophageal surgery or neck dissection.^[1]^ Moreover, there are no reports of transoral laser microsurgery (TLM) of the glottis causing tracheal rupture. The common complications of laser microsurgery for the glottis include loss of teeth, airway fire, post-surgical bleeding, dyspnea secondary to airway edema, pharyngeal bruising, aspiration pneumonia, cervical abscess, stenosis of the laryngeal vestibule, and thyroid cartilage chondritis.^[2]^ Tracheal rupture, as a rare and life-threatening complication, can easily be neglected by physicians, leading to serious consequences.

Here, we report a case of tracheal rupture after vocal cord polyp resection. This case report was approved by the Ethics Committee of the Affiliated Huashan Hospital, Fudan University. Informed written consent was obtained from the patient.

## 2 Case report

A 43-year-old woman, who presented with a 6-month history of hoarseness, was diagnosed with bilateral vocal cord polyps after laryngoscopy. Preoperative evaluation using chest computed tomography (CT) showed an intact tracheal wall with no abnormalities. Hence, vocal cord polyp resection via TLM was performed. Before the operation, a skilled anesthetist performed routine nasotracheal intubation with a video laryngoscope. The endotracheal tube cuff was filled with 10 mL water to replace air to make inadvertent laser damage to the endotracheal tube cuff more noticeable. During the operation, a saline-soaked absorbent cotton was placed at the subglottis to protect the trachea. There was no evidence of intraoperative damage to the tracheal wall.

**Figure 1:**
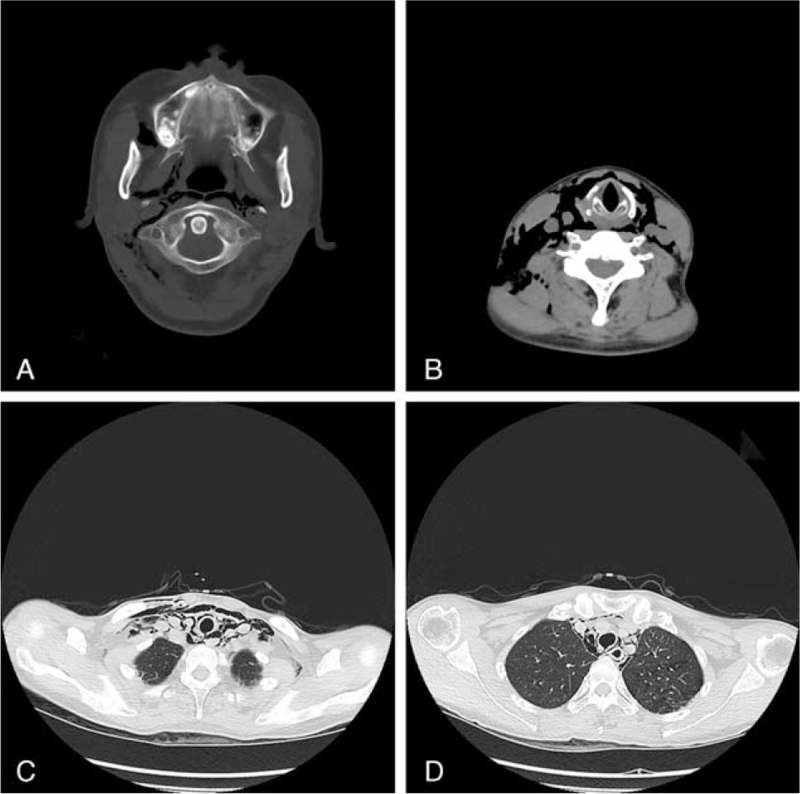
(A and B) The CT shows subcutaneous emphysema from the base of the skull to the chest; (C and D) tear on the tracheal wall from 4.5 to 8.5 cm under the glottis.

Fifteen hours after the operation, the patient experienced sudden dull pain in the anterior neck but no dyspnea. Repeat direct rigid laryngoscopy showed no abnormalities. This patient is considered to be a common throat in TML and discharged from the hospital. At 23 h after initial surgery, she returned to emergency room presenting subcutaneous emphysema and cough. Re-evaluation with a head, neck, and chest CT was done, which showed mediastinal emphysema and extensive subcutaneous emphysema from the base of the skull to the chest. A suspected tear on the membranous trachea located 4.5 cm under the glottis, extending to ∼8.5 cm under the glottis was noted (Fig. 1A–D). Bronchoscopy confirmed the tracheal rupture.

**Figure 2:**
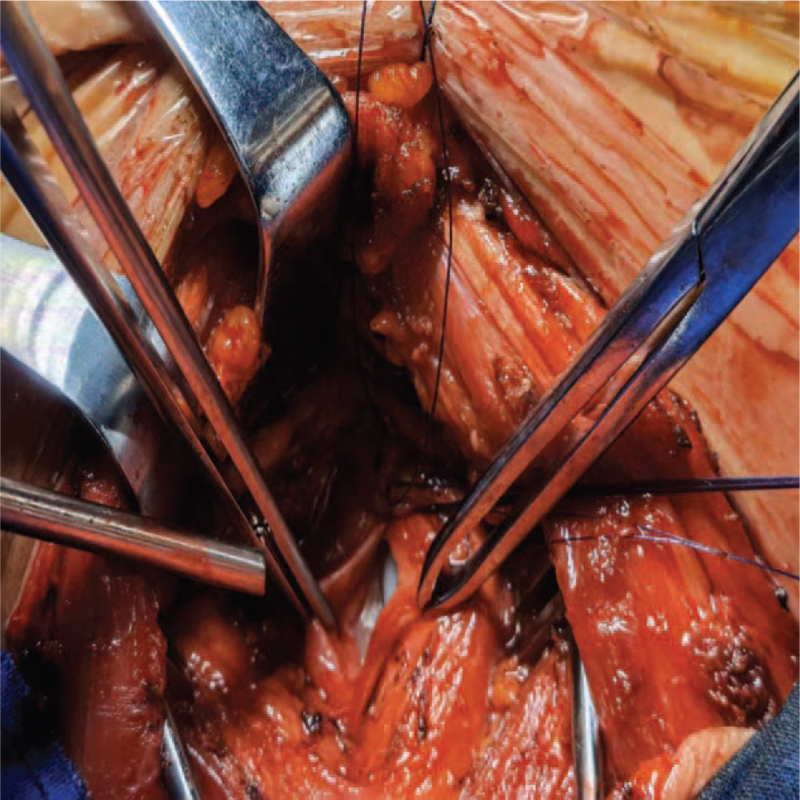
The chest exploration reveals the tear on tracheal wall.

The patient underwent emergency surgical chest exploration, which revealed a 4-cm long and narrow tracheal tear on the membranous trachea (Fig. 2). The tracheal tear was sutured, and the surgeon ensured that there were no other injuries.

The patient was discharged on the 11th postoperative day. During follow up after 1 month, no tracheal stenosis or respiratory dysfunction was noted.

## 3 Discussion

TLM has been widely used in pharyngeal and laryngeal surgery and offers several advantages in the treatment of glottic and supraglottic lesions,^[3]^ most of which are confined to the glottis and supraglottis. It is very rare to trigger tracheal rupture after TLM and most tracheal rupture are caused by intubation, tracheostomies, bronchoscopies, balloon dilation, or spontaneous tracheal rupture caused by coughing.^[4,5]^ Because we took adequate protective measures during the operation, we believed that tracheal rupture had no direct correlation with TLM. In our review of relevant literature, we excluded tracheal ruptures caused by bronchoscopies and tracheoscopies since these were not performed in our patient. For our case of tracheal rupture following TLM, two possible causes were considered:

1. Anatomical variations along with postoperative discomfort caused a cough that lead to spontaneous tracheal rupture and
2. Intubation caused injury to the trachea and overinflation of the balloon cuff might have caused an increased pressure on the tracheal wall.

Despite adequate measures to protect the trachea from laser injuries, TLM stimulation to the larynx may result in cough, leading to a high possibility of spontaneous tracheal rupture. Akkas et al. reported that predisposing factors of spontaneous tracheal rupture included anatomical variations such as that in chronic obstructive pulmonary disease, bronchial asthma, and tracheomalacia, as well as radiotherapy.^[5]^ Therefore, postoperative care, such as fog inhalation therapy, is particularly important and should be considered.

However, tracheal rupture usually does not occur even when high pressure is applied to the tracheal wall, due to the resiliency of the trachea.^[6]^ Therefore, the factors causing intubation injury also need to be considered. Several studies have found that multiple factors may lead to tracheal rupture following intubation, such as repetitive forceful attempts, overinflation of the balloon cuff, incorrect tube size, and improper tube positioning.^[7]^

The pharyngeal bruising is one of the most common postoperative complications of TLM.^[2]^ In this case, the patient's first clinical manifestations, neck dull pain, can be easily misdiagnosed as the pharyngeal bruising. Fortunately, the patient underwent timely chest CT to diagnose tracheal rupture and did not experience life-threatening complications such as shortness of breath and cyanosis. It is important to note that subcutaneous emphysema may not be present in the early period of tracheal rupture. Neck and chest CT are the gold standard methods for diagnosis, but these cannot be performed regularly. Therefore, patients with persistent postoperative pain after TLM and no obvious pharyngeal bruising should be treated with caution.

The optimal treatment for tracheal rupture remains controversial. In our case, the patient underwent an early surgical approach for tracheal repair and recovered 4 weeks after discharge. For patients in whom the lacerations smaller than 2 cm and do not have additional injuries, conservative treatment is more suitable.^[8]^

## 4 Conclusion

Tracheal rupture after TLM is a rare but easily overlooked and life-threatening complication and requires early diagnosis and accurate repair. The conventional protective measures and common postoperative complications in TLM may also cause tracheal rupture.

## Author contributions

**Conceptualization:** Xinqi Hu, Xidong Cui, Yitan Cao, Guangbin Sun.

**Data curation:** Xinqi Hu, Guangbin Sun.

**Formal analysis:** Xinqi Hu, Yitan Cao, Guangbin Sun.

**Investigation:** Xinqi Hu, Guangbin Sun.

**Methodology:** Xinqi Hu, Xiaofeng Chen, Xidong Cui, Guangbin Sun.

**Project administration:** Xinqi Hu, Xiaofeng Chen, Guangbin Sun.

**Resources:** Xinqi Hu, Guangbin Sun.

**Validation:** Xinqi Hu.

**Writing – original draft:** Xinqi Hu, Guangbin Sun.

**Writing – review & editing:** Xinqi Hu, Guangbin Sun.
